# Enhancement of tendon-to-bone healing after anterior cruciate ligament reconstruction using bone marrow-derived mesenchymal stem cells genetically modified with bFGF/BMP2

**DOI:** 10.1038/srep25940

**Published:** 2016-05-12

**Authors:** Biao Chen, Bin Li, Yong-Jian Qi, Qu-Bo Ni, Zheng-Qi Pan, Hui Wang, Liao-Bin Chen

**Affiliations:** 1 Department of Orthopaedic Surgery, Zhongnan Hospital, Wuhan University, Wuhan, China; 2 Department of Pharmacology, Basic Medical School, Wuhan University, Wuhan, China; 3 Hubei Provincial Key Laboratory of Developmentally Originated Disease, Wuhan, China

## Abstract

Many strategies, including various growth factors and gene transfer, have been used to augment healing after anterior cruciate ligament (ACL) reconstruction. The biological environment regulated by the growth factors during the stage of tendon-bone healing was considered important in controlling the integrating process. The purpose of this study was to evaluate the effects of bone marrow-derived mesenchymal stem cells (BMSCs) genetically modified with bone morphogenetic protein 2 (BMP2) and basic fibroblast growth factor (bFGF) on healing after ACL reconstruction. BMSCs were infected with an adenoviral vector encoding BMP2 (AdBMP2) or bFGF (AdbFGF). Then, the infected BMSCs were surgically implanted into the tendon-bone interface. At 12 weeks postoperatively, the formation of abundant cartilage-like cells, smaller tibial bone tunnel and significantly higher ultimate load and stiffness levels, through histological analysis, micro-computed tomography and biomechanical testing, were observed. In addition, the AdBMP2-plus-AdbFGF group had the smallest bone tunnel and the best mechanical properties among all the groups. The addition of BMP2 or bFGF by gene transfer resulted in better cellularity, new bone formation and higher mechanical property, which contributed to the healing process after ACL reconstruction. Furthermore, the co-application of these two genes was more powerful and efficient than either single gene therapy.

Anterior cruciate ligament (ACL) injuries lead to great morbidity in sports and daily life activities and may cause instability of the knee joint, leading to meniscus injuries and the subsequent development of degenerative joint disease^1^. Reconstruction surgery is chosen for ACL injuries in most cases because of the limited capacity of these injuries for self-healing. The pivotal healing process after ACL reconstruction largely depends on the integration of the graft with the host bone^2^,^3^,^4^. However, slow and incomplete healing of the tendon-to-bone interface may result in inferior functional rehabilitation and even worse osteoarthritic changes^5^,^6^,^7^. Thus, it is important to explore better strategies to accelerate and improve tendon-to-bone healing.

The gene transfer of therapeutic factors has been intensively developed to accelerate the early healing of the tendon-to-bone interface, which overcomes the limitations of the direct use of growth factors, including repeated applications and the short biological half-life of these proteins, and ensures a sustained delivery of growth factors at the injury site^8^. Basic fibroblast growth factor (bFGF) has been widely acknowledged for substantial roles in numerous cellular functions, including cell proliferation, angiogenesis, and tissue remodeling^9^,^10^,^11^,^12^. However, the effect of bFGF gene therapy for ACL repair remains unknown. To date, numerous studies have reported positive effects of bone morphogenetic protein 2 (BMP2) on healing after ACL reconstruction^13^,^14^. Kohno *et al.* reported that endogenous bFGF and BMP2 were both found at the tendon-to-bone interface after ACL reconstruction in a rabbit model^15^: in their findings, bFGF was expressed at the first 3 weeks of graft incorporation, but was absent at the 12 weeks, which contributed to fibrous integration between the tendon and bone via vascularization during the early postoperative phases. BMP2 was expressed throughout the 12-week study period, which was responsible for remodeling process of the bone, leading to osseous integration between the tendon and bone. Low levels of growth factors might be partly responsible for the slow or weak healing responses in the injured tendons. These discoveries indicated that a combination of these beneficial genes might have additive roles to achieve optimal efficacy in the molecular network system of the tendon-bone interface. Despite the promising role of bFGF and BMP2, there are very few studies that have assessed the effects of single growth factor bFGF on healing, or the functional implications of the combination bFGF-BMP2 for ACL reconstruction. Furthermore, our previous study demonstrated the superiority of using multiple growth factors via gene transfer to treat experimental osteoarthritis^16^. Thus, it was reasonable for us to hypothesize that sustained delivery of bFGF and BMP2 by gene transfer at the tendon-to-bone insertion site could achieve the best biological and biochemical effects on healing after ACL reconstruction.

The purpose of this study was to investigate the efficacy of promoting tendon-to-bone healing using bone marrow-derived mesenchymal stem cells (BMSCs) that were genetically modified with bFGF and BMP2. We hypothesized that transplanted cells genetically modified with either bFGF alone or the combination of BMP2 and bFGF would significantly enhance the healing process and that the combined growth factors would achieve the best effects.

## Results

### *In vitro* studies

#### Infection of BMSCs with adenovirus

**Figure 1:**
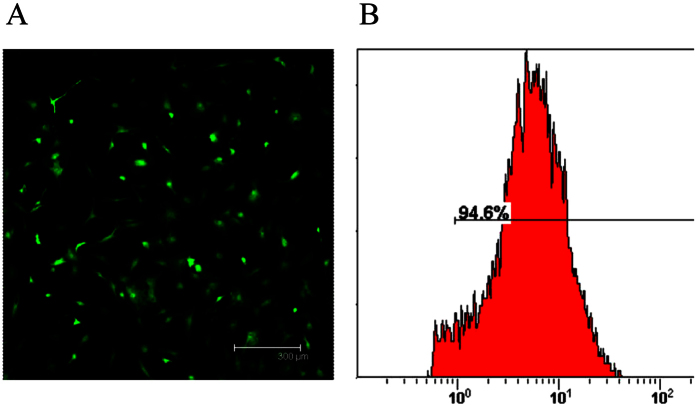
Determination of the adenoviral infection efficiency of BMSCs. (**A**) A photograph of transfected BMSCs was obtained under fluorescence microscopy. Scale: 300 μm. (**B**) The infection efficiency was quantified using flow cytometry.

To determine the optimal infection efficiency of BMSCs with the proper dosage of adenovirus, BMSCs were infected with adenovirus encoding enhanced green fluorescent protein (EGFP). After 72 h, a high efficiency of BMSCs with adenovirus at MOI 40 was observed via fluorescence microscopy (Fig. 1A). Furthermore, the percentage of EGFP-positive cells was 94.6% ± 3.24%, as demonstrated by flow cytometry analysis (Fig. 1B).

#### Gene expression levels and protein concentrations in the supernatants

**Figure 2:**
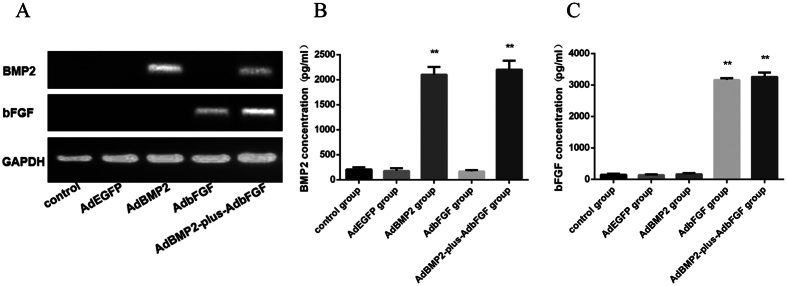
Adenovirus-mediated bFGF and BMP2 gene expression levels and protein concentrations in the supernatant. (**A**)The BMP2 and bFGF mRNA expression levels in transfected BMSCs at 72 h were analyzed by RT-PCR. The protein concentrations of BMP2 (**B**) and bFGF (**C**) were determined by ELISA. The bar represents the mean ± SEM of three independent experiments. *P < 0.05 and **P < 0.01 compared to the control group.

To identify whether the BMSCs successfully expressed the transfected genes of interest, the mRNA and protein levels of the target genes were detected after 3 days using RT-PCR and ELISA, respectively. RT-PCR demonstrated the specific expression of BMP2 in the AdBMP2 and AdBMP2-plus-AdbFGF groups and the specific expression of bFGF in the AdbFGF and AdBMP2-plus-AdbFGF groups (Fig. 2A). Furthermore, in accordance with the increased mRNA expression, the protein concentrations in the supernatant also increased. The concentrations of BMP2 were higher in the AdBMP2 group (2102.74 ± 154.03 pg/ml) and AdBMP2-plus-AdbFGF group (2201.97 ± 178.75 pg/ml) than in the control group (207.89 ± 40.23 pg/ml) and AdEGFP group (178.82 ± 53.30 pg/ml) (Fig. 2B). The concentrations of bFGF were higher in the AdbFGF group (3164.60 ± 52.00 pg/ml) and AdBMP2-plus-AdbFGF group (3257.35 ± 136.00 pg/ml) than in the control group (144.15 ± 31.22 pg/ml) and AdEGFP group (139.80 ± 18.77 pg/ml) (Fig. 2C).

#### Assay of cell viability

**Figure 3:**
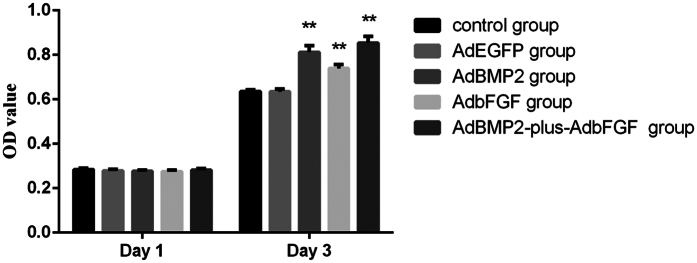
Analysis of viability of BMSCs using the MTS method. BMSCs were infected with various adenoviruses, after which their proliferation on days 1 and 3 was determined using the MTS assay. The bar represents the mean ± SEM of three independent experiments. *P < 0.05 and **P < 0.01.

To gain further insight into the viability of the adenovirus-infected BMSCs, cell proliferation was analyzed. There were no obvious differences among the groups in the viability of infected BMSCs on day 1 (Fig. 3), indicating that adenovirus infection had no detrimental effect at the indicated MOI. BMSCs proliferation was greatly enhanced in the AdBMP2, AdbFGF and AdBMP2-plus-AdbFGF groups compared to the control group after 3 days, whereas no difference was observed between the control and AdEGFP groups (Fig. 3).

### *In vivo* studies

#### Histological examination

**Figure 4:**
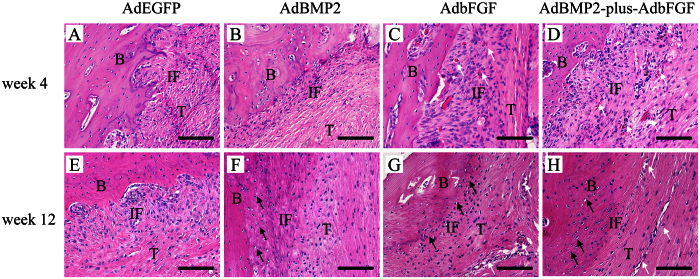
Histological evaluation of the tendon-bone interface by hematoxylin and eosin (H & E) staining at weeks 4 and 12 after surgery (200×). Black arrows mark the fibrocartilage-like cells, and white arrows mark the microvessels. The figure represents the histology of the tendon-to-bone interface in the AdEGFP, AdBMP2, AdbFGF and AdBMP2-plus-AdbFGF groups at both week 4 (**A–D**) and week 12 (**E–H**) after surgery. B: bone; T: tendon; IF: interface. Scale: 25 μm.

Histological images obtained at 4 weeks after surgery revealed no evidence of acute inflammatory response to the adenoviral vectors at the tendon-to-bone interface. In the AdEGFP group, the histology of most regions showed some disorderly arranged porous fibrous tissues between the tendon and bone tunnel (Fig. 4A). In the AdBMP2 group, a narrow zone of newly formed matrix resembling chondro-osteoid was seen along the bone tunnel (Fig. 4B). In the AdbFGF group, the formation of a broad fibrovascular interface and perpendicular collagen fibers along the load axis was observed, followed by newly formed vessels in the graft substance (Fig. 4C). In the AdBMP2-plus-AdbFGF group, a broad zone of newly formed matrix resembling chondro-osteoid, as well as some large vessels, was noted at the interface (Fig. 4D).

At 12 weeks postsurgery, a gradual reestablishment of collagen fiber continuity perpendicular to the load axis between the tendon and bone was noted in the AdEGFP group (Fig. 4E). A transition from tendon to non-mineralized fibrocartilage and mineralized cartilage was observed in the AdBMP2 (Fig. 4F), AdbFGF (Fig. 4G), and AdBMP2-plus-AdbFGF (Fig. 4H) groups. Additionally, the AdBMP2-plus-AdbFGF group had a broader area of cartilage-like cells than the AdBMP2 or AdbFGF group. Moreover, some microvessels were found at the interface in the AdbFGF and AdBMP2-plus-AdbFGF groups.

#### Micro-CT analysis

**Figure 5:**
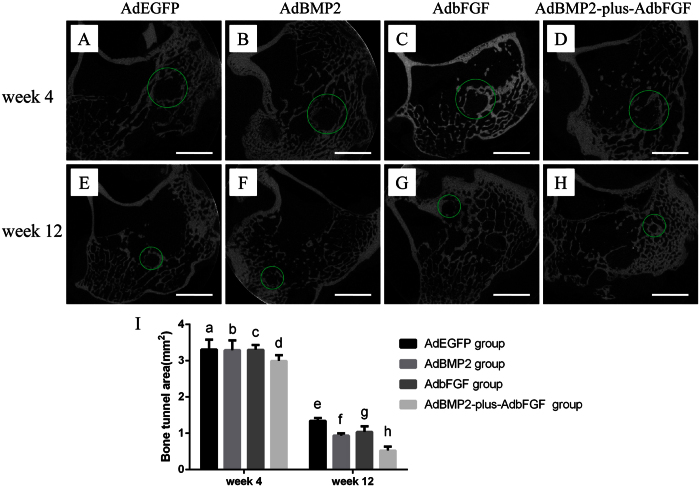
Micro-CT image of each group at 4 and 12 weeks postoperatively. Green circles mark the bone tunnel. The areas of the bone tunnel were scanned among the AdEGFP, AdBMP2, AdbFGF, and AdBMP2-plus-AdbFGF groups at both 4 weeks (**A–D**) and 12 weeks (**E–H**). Quantitative analysis of the bone tunnel areas was performed at both 4 and 12 weeks (**I**). The bar represents the mean ± SEM, n = 6. At 4 weeks, there was no difference among the groups. At 12 weeks, f vs. e, P = 0.04; f vs. g, P = NS; g vs. e, P = 0.1; h vs. e, P < 0.01; h vs. f, P = 0.04; and h vs. g, P = 0.01.NS = no significance. Scale: 5 mm.

The mean areas of the bone tunnel in the AdEGFP, AdBMP2, AdbFGF and AdBMP2-plus-AdbFGF groups were 3.31 ± 0.27 mm^2^, 3.29 ± 0.27 mm^2^, 3.30 ± 0.13 mm^2^ and 2.99 ± 0.16 mm^2^, respectively, at 4weeks (Fig. 5A–D) and 1.34 ± 0.08 mm^2^, 0.94 ± 0.06 mm^2^, 1.04 ± 0.16 mm^2^ and 0.53 ± 0.10 mm^2^, respectively, at 12 weeks (Fig. 5E–H). There was no significant difference among the groups at 4 weeks. At 12 weeks, the area of the AdbFGF group showed a decreased tendency (P = 0.1), but the areas of the AdBMP2 and AdBMP2-plus-AdbFGF groups were significantly smaller than that of the AdEGFP group. In addition, the AdBMP2-plus-AdbFGF group exhibited a substantially smaller area than the AdBMP2 and AdbFGF groups, but there was no significant difference between the AdBMP2 and AdbFGF groups (Fig. 5I).

#### Mechanical evaluation

**Figure 6:**
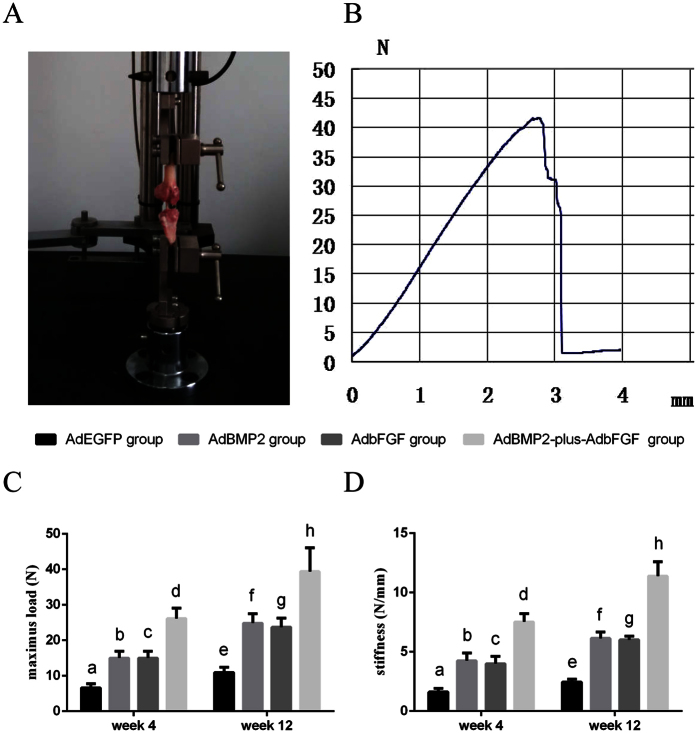
Mechanical examination of graft-bone healing in a rabbit model at 4 and 12 weeks after surgery. (**A**) The femur-graft-tibia was mounted in the clamps, and mechanical testing was performed. (**B**) A typical load-displacement curve was generated from the biomechanical test. (**C**) Maximum load at 4 and 12 weeks. (**D**) Stiffness at 4 and 12 weeks. The bar represents the mean ± SEM, n = 6. Maximum load: Week 4: b vs. a, P = 0.03; b vs. c, P = NS; c vs. a, P = 0.04; d vs. a, P < 0.01; d vs. b, P < 0.01; d vs. c, P < 0.01. Week 12: f vs. e, P = 0.04; f vs. g, P = NS; g vs. e, P = 0.04; h vs. e, P < 0.01; h vs. f, P = 0.03; h vs. g, P = 0.02. Stiffness: Week 4: b vs. a, P = 0.01; b vs. c, P = NS; c vs. a, P = 0.02; d vs. a, P < 0.01; d vs. b, P < 0.01; d vs. c, P < 0.01. Week 12: f vs. e, P = 0.03; f vs. g, P = NS; g vs. e, P = 0.03; h vs. e, P < 0.01; h vs. f, P < 0.01; h vs. g, P < 0.01. NS = no significance.

The mean values of the maximum load of grafted-tendon failure in the AdEGFP, AdBMP2, AdbFGF and AdBMP2-plus-AdbFGF groups were 6.59 ± 1.15 N, 15.01 ± 1.94 N, 14.16 ± 1.64 N and 26.16 ± 2.89 N, respectively, at 4 weeks and 12.25 ± 2.49 N, 21.85 ± 2.76 N, 21.16 ± 2.84 N and 39.39 ± 6.72 N, respectively at 12 weeks (Fig. 6C). The maximum loads of the AdBMP2, AdbFGF and AdBMP2-plus-AdbFGF groups were significantly higher than that of the AdEGFP group. In addition, the AdBMP2-plus-AdbFGF group exhibited a substantially higher maximum load than the AdBMP2 and AdbFGF groups, but there was no significant difference between the AdBMP2 and AdbFGF groups at either 4 or 12 weeks.

The mean stiffness values of the reconstructed ACLs in the AdEGFP, AdBMP2, AdbFGF and AdBMP2-plus-AdbFGF groups were 1.62 ± 0.31 N/mm, 4.26 ± 0.64 N/mm, 4.00 ± 0.62 N/mm, and 7.53 ± 0.69 N/mm, respectively, at 4 weeks and 2.64 ± 0.24 N/mm, 6.15 ± 0.53 N/mm, 6.01 ± 0.32 N/mm and 11.39 ± 1.22 N/mm, respectively, at 12 weeks (Fig. 6D). The stiffness values of the AdBMP2, AdbFGF and AdBMP2-plus-AdbFGF groups were dramatically greater than that of the AdEGFP group. In addition, the AdBMP2-plus-AdbFGF group displayed considerably greater stiffness than the AdBMP2 and AdbFGF groups, but there was no significant variation between the AdBMP2 and AdbFGF groups at either 4 or12 weeks.

## Discussion

To our knowledge, this report is the first demonstration of the therapeutic potential of gene therapy using adenoviral-mediated BMSCs expressing BMP2 and bFGF for healing after ACL reconstruction. The healing effect was due to the formation of cartilage-like cells at the interface, a smaller bone tunnel area and stronger ultimate load and stiffness. Furthermore, the effects of combinatorial BMP2 and bFGF gene therapy were more powerful than those of single gene therapy in accelerating tendon-to-bone healing. These findings suggest the potential of the co-application of growth factors in gene therapy to biologically and functionally improve the regeneration of the tendon-to-bone interface.

There were two types of tendon-to-bone insertions about the healing of ACL reconstruction: indirect and direct insertions. In indirect insertions, the graft directly contacts the bone tunnel through Sharpey’s fibers, whereas direct insertions are characterized by a layered chondral formation at the graft-bone interface^17^. In the AdbFGF group, we noted a broad fibrovascular interface at 4 weeks, followed by the formation of cartilage-like cells at 12 weeks, suggesting a shift from an immature to a mature state of tissue regeneration over time. Tozer *et al.* indicated that bFGF was one of best-characterized growth factors for tissue repair, and its major action was promotion of collagen production, tendon development, and proliferation of tenocytes^18^. Kohno *et al.* found that bFGF contributed to fibrous integration between the tendon and bone via vascularization during the early postoperative phases^15^. Besides, Tang *et al.* revealed that the introduction of bFGF gene to the tendon could promote expression of a series of growth factor genes, such as transforming growth factor (TGF) β1, vascular endothelial growth factor (VEGF), and connective tissue growth factor within the tendon healing period^19^. Moreover, Steinert *et al.* demonstrated that ACL cells expressed stem cell markers and were able to undergo multi-directional differentiation^20^. Haddad-Weber *et al.* revealed that ACL fibroblasts were more efficient for induction differentiation than mesenchymal stem cells^21^. Consequently, the histological effects of bFGF on tendon-to-bone healing in the present study seem to be predominantly attributable to stimulation of the proliferation and cartilage-like differentiation of mesenchymal cells as well as fibroblasts of the grafts, in which bFGF might act as a factor initiating a cascade of events to enhance the release of additional factors. Meanwhile, we also observed some micro-vessles at the interface at 4 weeks and in the substance of the graft at 12 weeks. Mifune *et al.* reported that the transplanted ACL-derived CD34 + cells could promote angiogenesis not only in the outside of the grafts but also the interior of the grafted tendon due to the migration, endothelial differentiation and the VEGF secretion^22^. Woad *et al.* showed that bFGF could promote endothelial cells migration, proliferation and induce vascular sprouting^9^. Thus, we considered that endothelial differentiation and bFGF secretion appeared to be key mechanism for promoting angiogenesis in our findings.

In the AdBMP2 group, the histological analysis showed a zone of newly formed matrix resembling chondro-osteoid at week 4 and cartilage-like cells at week 12 at the tendon-to-bone interface. Martinek *et al*. found similar results using BMP2 gene transfer in a tendon-to-bone healing model^23^. These findings correlated closely with the biological role of BMP2 as a stimulus, suggesting that BMP2 might increase the proliferation of implanted BMSCs, induce differentiation into fibrocartilage-like cells and promote the shift from non-mineralized fibrocartilage to mineralized cartilage.

Not only was the local tissue at the interface examined microscopically, but the newly formed mineralized tissue was also analyzed to discern the subtle changes. In order to show higher sensitivity for bone tunnel measurement during early stage of reconstruction, micro-CT was used to evaluate the postoperative bone tunnel, which ensured the accuracy and precision of the location of a series of bone tunnel cross sections by coronal, sagittal and axial inspection, without any postural variation^24^,^25^,^26^. The micro-CT evaluation at 4 weeks showed that although the bone tunnel areas exhibited no obvious differences among the groups, they were smaller in the AdBMP2 and AdbFGF groups at 12 weeks. These findings are consistent with the report of Sasaki *et al.*^24^, who noted using micro-CT analysis that the tibial bone tunnel was significantly smaller in the granulocyte colony-stimulating factor group due to the differentiation and activation of osteoblasts accumulated at the bone tunnel surface. Martinek *et al.* also reported similar results: BMP2 gene transfer significantly improved the integration of semitendinosus tendon grafts in bone tunnels by increasing osteoblast activation in the formation of a chondro-osteoid matrix^23^. Both bFGF and BMP2 can promote the differentiation of BMSCs into osteoblasts and can promote osteoblast activity^27^,^28^,^29^,^30^. Regarding our results, a possible reason for these changes might be that both bFGF and BMP2 increased the activity of osteoblasts and promoted tissue remolding and mineralization at the tendon-to-bone interface.

According to the biomechanical assessment, the ultimate load and stiffness of the AdbFGF and AdBMP2 groups was increased at 4 weeks and 12 weeks. In our opinion, the improved mechanical properties at 4 weeks correlated with the histological changes, which allowed for the lack of obvious bone tunnel ingrowth observed by micro-CT at this time point. This finding suggests that during the early healing period, the establishment and arrangement of collagen fiber continuity between the graft and bone tunnel could promote mechanical strength. Subsequently, the differentiation of local cells, tissue remolding and accompanying osseous ingrowth at 12 weeks contributed to the stronger mechanical properties. Other studies have reported parallel results regarding the mechanical properties of grafts after ACL reconstruction^13^,^26^,^31^.

Notably, the AdBMP2-plus-AdbFGF group exhibited the broadest zone resembling the native insertion of the tendon-to-bone interface and the smallest bone tunnel area at 12 weeks, as well as the greatest mechanical strength, suggesting synergistic effects of BMP2 and bFGF on the healing process. Our results parallel those of Wang *et al.*, who claimed that the combination of BMP2 and bFGF was more effective than either factor alone at promoting the formation of new bone^32^. It has been reported that BMP2 can also induce bFGF expression *in vivo*^33^ and that the combination of BMP2 and bFGF could significantly promote BMSCs to differentiate into osteoblasts and promote osteoblast activity^30^,^34^. Hence, a possible mechanism mediating the synergy in our findings might be as follows: first, BMP2 and bFGF could enhance the synthesis of each other. Then, BMP2 and bFGF might promote the proliferation and differentiation of both BMSCs and cells derived from the surrounding marrow cavity. Finally, bFGF and BMP2 could facilitate the maturation and mineralization of collagen fibers and the remodeling of the bone tunnel to advance biological integration at the interface. The superiority of two growth factors by gene transfer was also demonstrated by Wei *et al.*, who reported that the co-expression of TGF β1/VEGF165 was more efficient and powerful than single gene therapy in promoting the healing of reconstructed ACL^35^.

Our study has several limitations. First, this preliminary study was conducted to evaluate the ability of growth factors to accelerate early healing because constructed grafts are liable to fail at the tendon-to-bone attachment site. However, it will be necessary in the future to observe whether the histological and biomechanical differences observed among the groups remain the same or diminish over time. Furthermore, the precise mechanisms by which these two growth factors regulate healing after ACL reconstruction have not been elucidated.

In conclusion, our results reveal that BMSCs genetically modified with bFGF could augment healing after ACL reconstruction. Furthermore, the synergistic effects of BMP2 and bFGF were greater than that of a single gene during this process. Based on the present study’s thorough investigation of the biological healing process after ACL reconstruction, we firmly believe that suitable growth factors that are capable of transducing a series of signals in a distinct spatial and temporal pattern may recreate the structure and composition of the native direct tendon-to-bone interface, which provides a promising strategy for future studies.

## Material and Methods

### *In vitro* studies

#### Cell isolation and culture

Four healthy, adult male New Zealand white rabbits (average weight, 1.0 kg) were purchased from the Experimental Animal Department of Hubei Provincial Center for Disease Control and Prevention. After the rabbits were anesthetized, the femur and tibia were harvested and removed metaphysis of both sides with rongeur under sterile condition. Bone marrow from all donors was mixed and collected together by flushing the femur and tibia with medium and centrifuging the flushed liquid at 1200 rpm for 8 min. The supernatants were discarded, and the cell pellets were resuspended in culture medium containing DMEM/F12 (Invitrogen, USA), 10% fetal bovine serum (Gibco, USA) and 100 units/ml penicillin-streptomycin, then followed by culture in a humidified 5% CO_2_ atmosphere at 37 °C. The medium was changed every 3 days. After proliferation, the adhesive cells, which were defined as BMSCs using a protocol described previously^36^, were subcultured to passage 3 for the following experiments.

#### Construction and infection of recombinant adenoviral vectors

To obtain adenoviral bFGF and BMP2, the entire human coding sequences of bFGF (480bp) and BMP2 (1.2 kb) were inserted into an adenoviral plasmid containing EGFP under the control of the cytomegalovirus (CMV) promoter. The produced vectors were designated AdbFGF and AdBMP2, respectively, and were reproduced according to procedures described in our previous study. The optimal multiplicity of infection (MOI) value was determined for the adenoviral infection of BMSCs in a preliminary experiment. Briefly, BMSCs were seeded and infected with adenoviral vectors at various MOIs. After 48 h, flow cytometry was used to identify the EGFP-expressing infected cells, which were captured at the optimal MOI under an inverted fluorescence microscope.

#### Real-time PCR analysis and enzyme-linked immunosorbent assay (ELISA)

Total RNA was extracted from the cells 72 h after viral infection using Trizol reagent (Invitrogen, USA) following the manufacturer’s instructions, and the RNA was converted to cDNA using the PrimeScript® RT reagent kit (Takara Biotechnology Co., Ltd., Dalian, China). The BMP2 PCR primer sequences were as follows: forward AGTGGGTGCTGCTCTTCCTA and reverse ATGGGACACTCCTCTGTTGG. The bFGF PCR primer sequences were as follows: forward GTGTTACGGATGAGTGTTTCT and reverse CAGCTCTTAGCAGACATTGG. The housekeeping gene glyceraldehyde 3-phosphate dehydrogenase (GAPDH) was used as an internal reference, and its primer sequences were as follows: forward ACGGATTTGGTCGTATTGGG and reverse TGATTTTGGAGGGATCTCGC. The PCR products were subjected to electrophoresis on 1.5% agarose gels. The cell supernatants were collected after infected BMSCs were cultured for 72 h. The concentrations of bFGF and BMP2 in the cell supernatants were determined using ELISA kits (R&D Systems, Minneapolis, MN, USA) according to the manufacturer’s protocols.

#### Assay of cell viability

A cell proliferation assay was performed using tetrazolium compound based CellTiter 96^®^Aqueous One Solution Cell Proliferation (MTS) assay (Promega, Shanghai, China). BMSCs were seeded into wells of a 96-well plate at a density of 4 × 10^3^ and infected at the indicated MOI with adenoviruses. After 1 and 3 days of culture, a MTS assay was performed according to the manufacturer’s instructions. Each experiment was performed and repeated 3 times.

### *In vivo* studies

#### Animal study design

Thirty-two healthy, mature New Zealand white rabbits weighing an average of 2.5 kg (2.0–3.0 kg) were purchased from the Laboratory Animal Center of Wuhan University (Wuhan, Hubei, China). All of the following procedures were approved by and performed in accordance with the guidelines of the Ethical and Research Committee of the Medical College of Wuhan University. Both hind legs of each rabbit were used. ACLs were reconstructed with the semitendinosus, and approximately 1 × 10^7^ BMSCs that had been infected with the recombinant EGFP, BMP2, bFGF or BMP2-plus-bFGF adenovirus and immobilized in 0.2 ml of fibrin glue (Puji Biotechnology, Hangzhou, China) were injected into the tendon-to-bone interface. Based on the different BMSC treatments, the rabbits were randomly allocated to four groups (8 rabbits per group): the (1) AdEGFP, (2) AdBMP2, (3) AdbFGF, and (4) AdBMP2-plus-AdbFGF groups. Specimens were harvested from each group at 4 and 12 weeks postoperatively for biomechanical and histological analyses.

#### Surgical procedure

The rabbit model of ACL reconstruction was established according to the protocols previously reported by Lim *et al.*^37^,^38^. Briefly, after anesthesia with an intravenous injection of 3% pentobarbital (30 mg/kg), the animals were fixed on the operation table in the supine position with their knees being able to flex freely to 90°. Both of the hind legs were shaved and aseptically prepared for surgery. A 4-cm-long medial parapatellar incision was performed to expose the knee joint. Patella was dislocated laterally and infrapatella fat pad was then removed to expose the joint cavity. ACL was excised from its femoral and tibial insertions. A 2.5-mm-diameter drill was used to create the femur and tibia tunnels through the original footprints of the ACL. The semitendinosus was dissected distally at the musculotendinous junction. After a weaving suture using 2-0 Ethibond (Ethicon, Shanghai, China), the graft was passed through the tunnels. The tunnels were irrigated with normal saline before application of experimental agents. A total of 1 × 10^7^ BMSCs that had been treated with corresponding adenoviruses one day prior and then immobilized in 0.2 ml of fibrin glue were evenly injected into the tendon-to-bone interface of each leg in rabbits of the four groups with a micro-syringe. The distal end of the tendon was then sutured to the periosteum using 2-0 Ethibond (Ethicon, Shanghai, China) on the femoral side with the knee at 30°of flexion. The wound was closed in layers. Postoperatively, the animal was returned to the housing cage and allowed to perform general activities. A prophylactic antibiotic (40 mg/kg penicillin) was administered intramuscularly for 7 days.

#### Histological examination

Hematoxylin and eosin (H&E) staining was performed to evaluate the healing capacity of the tendon-to-bone interface after ACL reconstruction. The specimens were removed from the animal immediately after sacrifice. After removing the intra-articular graft, the intraosseous graft-bone complex was kept and fixed in 4% paraformaldehyde in a 0.1 M phosphate-buffered solution for 72 h. Then, each sample was decalcified in 0.5 M ethylenediaminetetraacetic acid, dehydrated in a graded series of ethanol, and embedded in paraffin. The samples were sectioned using a microtome (Leica Instrument Co., Ltd., Shanghai, China) parallel to the longitudinal axis of the tibial tunnel with a thickness of 5 μm. These sections were stained with H&E, and the tendon-to-bone interface was examined under light microscopy (Olympus, Tokyo, Japan).

#### Micro-CT analysis

Using multiplanar reconstruction, micro-computed tomography (CT) (μCT-40, Scanco Medical, Brüttisellen, Switzerland) images were obtained to assess multiple sections of bone tunnel. The examination protocol was performed as previously described^24^,^26^. The areas of the vertical plane across the axis of the bone tunnel were measured at a depth of 5 mm from the tibial joint surface. The X-ray levels were 70 kV and 114 μA. Each area was measured three times with image analysis software (ImageJ; National Institutes of Health), and the average value was used for analysis.

#### Biomechanical testing

Knee samples with the femur and tibia kept at a length of 50 mm from the joint were harvested from the animal immediately after sacrifice and frozen at −80 °C until testing. The specimens were thawed overnight at room temperature, and then the femur–ACL graft–tibia complex was separated by resecting the attached soft tissue. The sutures that were used to fix the grafts were also removed before testing. Biomechanical testing was performed using a materials testing machine (805, Instron Co., Norwood, MA, USA). The complex was fixed between the U-shaped clamps with 45° of knee flexion to ensure that the pulling force was parallel to the axis of the graft, with a displacement rate of 10 mm/min. The ultimate failure load and stiffness were determined from the load-displacement curve.

#### Statistical Analysis

The results of the quantitative assays were expressed as the means ± standard error of the mean (SEM). A one-way analysis of variance (one-way ANOVA) was used to compare differences in the quantitative results from the groups at weeks 4 and 12, followed by a post-hoc Holm-Sidak test using SPSS 17 (SPSS Science Inc., Chicago, Illinois) to determine which two of the four groups differed significantly. P < 0.05 was considered statistically significant.

## Additional Information

**How to cite this article**: Chen, B. *et al.* Enhancement of tendon-to-bone healing after anterior cruciate ligament reconstruction using bone marrow-derived mesenchymal stem cells genetically modified with bFGF/BMP2. *Sci. Rep.* **6**, 25940; doi: 10.1038/srep25940 (2016).

## References

[b1] von Porat A., Roos E. M. & Roos H. High prevalence of osteoarthritis 14 years after an anterior cruciate ligament tear in male soccer players: a study of radiographic and patient relevant outcomes. Ann Rheum Dis 63, 269–273 (2004). 14962961 10.1136/ard.2003.008136 PMC1754918

[b2] Goradia V. K., Rochat M. C., Grana W. A., Rohrer M. D. & Prasad H. S. Tendon-to-bone healing of a semitendinosus tendon autograft used for ACL reconstruction in a sheep model. Am J Knee Surg 13, 143–151 (2000). 11277242

[b3] Papageorgiou C. D., Ma C. B., Abramowitch S. D., Clineff T. D. & Woo S. L. A multidisciplinary study of the healing of an intraarticular anterior cruciate ligament graft in a goat model. Am J Sports Med 29, 620–626 (2001). 11573921 10.1177/03635465010290051501

[b4] Kurosaka M., Yoshiya S. & Andrish J. T. A biomechanical comparison of different surgical techniques of graft fixation in anterior cruciate ligament reconstruction. Am J Sports Med 15, 225–229 (1987). 3303979 10.1177/036354658701500306

[b5] Lohmander L. S., Englund P. M., Dahl L. L. & Roos E. M. The long-term consequence of anterior cruciate ligament and meniscus injuries: osteoarthritis. Am J Sports Med 35, 1756–1769 (2007). 17761605 10.1177/0363546507307396

[b6] Oiestad B. E., Engebretsen L., Storheim K. & Risberg M. A. Knee osteoarthritis after anterior cruciate ligament injury: a systematic review. Am J Sports Med 37, 1434–1443 (2009). 19567666 10.1177/0363546509338827

[b7] Lohmander L. S., Ostenberg A., Englund M. & Roos H. High prevalence of knee osteoarthritis, pain, and functional limitations in female soccer players twelve years after anterior cruciate ligament injury. Arthritis Rheum 50, 3145–3152 (2004). 15476248 10.1002/art.20589

[b8] Lattermann C. *et al.* Gene transfer to the tendon-bone insertion site. Knee Surg Sports Traumatol Arthrosc 12, 510–515 (2004). 15014945 10.1007/s00167-003-0482-4

[b9] Woad K. J. *et al.* Fibroblast growth factor 2 is a key determinant of vascular sprouting during bovine luteal angiogenesis. Reproduction 143, 35–43 (2012). 21998077 10.1530/REP-11-0277

[b10] Fei Y., Xiao L., Doetschman T., Coffin D. J. & Hurley M. M. Fibroblast growth factor 2 stimulation of osteoblast differentiation and bone formation is mediated by modulation of the Wnt signaling pathway. J Biol Chem 286, 40575–40583 (2011). 21987573 10.1074/jbc.M111.274910 PMC3220493

[b11] Tsurushima H. *et al.* Enhanced bone formation using hydroxyapatite ceramic coated with fibroblast growth factor-2. Acta Biomater 6, 2751–2759 (2010). 20045091 10.1016/j.actbio.2009.12.045

[b12] Chen M. *et al.* Roles of exogenously regulated bFGF expression in angiogenesis and bone regeneration in rat calvarial defects. Int J Mol Med 27, 545–553 (2011). 21327326 10.3892/ijmm.2011.619

[b13] Wang C. J. *et al.* pCMV-BMP-2-transfected cell-mediated gene therapy in anterior cruciate ligament reconstruction in rabbits. Arthroscopy 26, 968–976 (2010). 20620796 10.1016/j.arthro.2009.11.014

[b14] Dong Y., Zhang Q., Li Y., Jiang J. & Chen S. Enhancement of tendon-bone healing for anterior cruciate ligament (ACL) reconstruction using bone marrow-derived mesenchymal stem cells infected with BMP-2. Int J Mol Sci 13, 13605–13620 (2012). 23202970 10.3390/ijms131013605 PMC3497344

[b15] Kohno T. *et al.* Immunohistochemical demonstration of growth factors at the tendon-bone interface in anterior cruciate ligament reconstruction using a rabbit model. J Orthop Sci 12, 67–73 (2007). 17260120 10.1007/s00776-006-1088-8

[b16] Chen B., Qin J., Wang H., Magdalou J. & Chen L. Effects of adenovirus-mediated bFGF, IL-1Ra and IGF-1 gene transfer on human osteoarthritic chondrocytes and osteoarthritis in rabbits. Exp Mol Med 42, 684–695 (2010). 20733349 10.3858/emm.2010.42.10.067 PMC2966742

[b17] Park M. J., Lee M. C. & Seong S. C. A comparative study of the healing of tendon autograft and tendon-bone autograft using patellar tendon in rabbits. Int Orthop 25, 35–39 (2001). 11374265 10.1007/s002640000199 PMC3620615

[b18] Tozer S. & Duprez D. Tendon and ligament: development, repair and disease. Birth Defects Res C Embryo Today 75, 226–236 (2005). 16187327 10.1002/bdrc.20049

[b19] Tang J. B., Chen C. H., Zhou Y. L., McKeever C. & Liu P. Y. Regulatory effects of introduction of an exogenous FGF2 gene on other growth factor genes in a healing tendon. Wound Repair Regen 22, 111–118 (2014). 24393159 10.1111/wrr.12129

[b20] Steinert A. F. *et al.* Mesenchymal stem cell characteristics of human anterior cruciate ligament outgrowth cells. Tissue Eng Part A 17, 1375–1388 (2011). 21247268 10.1089/ten.tea.2010.0413 PMC3079172

[b21] Haddad-Weber M. *et al.* BMP12 and BMP13 gene transfer induce ligamentogenic differentiation in mesenchymal progenitor and anterior cruciate ligament cells. Cytotherapy 12, 505–513 (2010). 20334610 10.3109/14653241003709652 PMC3580941

[b22] Mifune Y. *et al.* Tendon graft revitalization using adult anterior cruciate ligament (ACL)-derived CD34 + cell sheets for ACL reconstruction. Biomaterials 34, 5476–5487 (2013). 23632324 10.1016/j.biomaterials.2013.04.013

[b23] Martinek V. *et al.* Enhancement of tendon-bone integration of anterior cruciate ligament grafts with bone morphogenetic protein-2 gene transfer: a histological and biomechanical study. J Bone Joint Surg Am 84-A, 1123–1131 (2002). 12107310 10.2106/00004623-200207000-00005

[b24] Sasaki K. *et al.* Enhancement of tendon-bone osteointegration of anterior cruciate ligament graft using granulocyte colony-stimulating factor. Am J Sports Med 36, 1519–1527 (2008). 18413678 10.1177/0363546508316282

[b25] Zhang X. *et al.* Runx2-Modified Adipose-Derived Stem Cells Promote Tendon Graft Integration in Anterior Cruciate Ligament Reconstruction. Sci Rep 6, 19073 (2016). 26743583 10.1038/srep19073 PMC4705474

[b26] Bi F., Shi Z., Liu A., Guo P. & Yan S. Anterior cruciate ligament reconstruction in a rabbit model using silk-collagen scaffold and comparison with autograft. PLoS One 10, e0125900 (2015). 25938408 10.1371/journal.pone.0125900 PMC4418759

[b27] Rose L. C. *et al.* Effect of basic fibroblast growth factor in mouse embryonic stem cell culture and osteogenic differentiation. J Tissue Eng Regen Med 7, 371–382 (2013). 22674886 10.1002/term.532

[b28] Hikiji H. *et al.* An *in vivo* murine model for screening cranial bone regenerative materials: testing of a novel synthetic collagen gel. J Mater Sci Mater Med 25, 1531–1538 (2014). 24573457 10.1007/s10856-014-5185-5

[b29] Kuhn M. C. *et al.* Adipocyte-secreted factors increase osteoblast proliferation and the OPG/RANKL ratio to influence osteoclast formation. Mol Cell Endocrinol 349, 180–188 (2012). 22040599 10.1016/j.mce.2011.10.018

[b30] Li P. *et al.* Synergistic and sequential effects of BMP-2, bFGF and VEGF on osteogenic differentiation of rat osteoblasts. J Bone Miner Metab 32, 627–635 (2014). 24306516 10.1007/s00774-013-0538-6

[b31] Dong S. *et al.* Decellularized Versus Fresh-Frozen Allografts in Anterior Cruciate Ligament Reconstruction: An *In Vitro* Study in a Rabbit Model. Am J Sports Med 43, 1924–1934 (2015). 26037623 10.1177/0363546515585314

[b32] Wang L. *et al.* Repair of bone defects around dental implants with bone morphogenetic protein/fibroblast growth factor-loaded porous calcium phosphate cement: a pilot study in a canine model. Clin Oral Implants Res 22, 173–181 (2011). 20678131 10.1111/j.1600-0501.2010.01976.x

[b33] Alam S. *et al.* Expression of bone morphogenetic protein 2 and fibroblast growth factor 2 during bone regeneration using different implant materials as an onlay bone graft in rabbit mandibles. Oral Surg Oral Med Oral Pathol Oral Radiol Endod 103, 16–26 (2007). 17178489 10.1016/j.tripleo.2006.01.019

[b34] Draenert F. G., Nonnenmacher A. L., Kammerer P. W., Goldschmitt J. & Wagner W. BMP-2 and bFGF release and *in vitro* effect on human osteoblasts after adsorption to bone grafts and biomaterials. Clin Oral Implants Res 24, 750–757 (2013). 22524399 10.1111/j.1600-0501.2012.02481.x

[b35] Wei X. *et al.* Local administration of TGFbeta-1/VEGF165 gene-transduced bone mesenchymal stem cells for Achilles allograft replacement of the anterior cruciate ligament in rabbits. Biochem Biophys Res Commun 406, 204–210 (2011). 21303664 10.1016/j.bbrc.2011.02.015

[b36] Deng Y. *et al.* Effect of nicotine on chondrogenic differentiation of rat bone marrow mesenchymal stem cells in alginate bead culture. Biomed Mater Eng 22, 81–87 (2012). 22766705 10.3233/BME-2012-0692

[b37] Lim J. K. *et al.* Enhancement of tendon graft osteointegration using mesenchymal stem cells in a rabbit model of anterior cruciate ligament reconstruction. Arthroscopy 20, 899–910 (2004). 15525922 10.1016/j.arthro.2004.06.035

[b38] Ouyang H. W. Use of Bone Marrow Stromal Cells for Tendon Graft-to-Bone Healing: Histological and Immunohistochemical Studies in a Rabbit Model. Am J Sports Med 32, 321–327 (2004). 14977654 10.1177/0095399703258682

